# Pilot phase evaluation of the elective general practice class: results of student surveys of the first two years

**DOI:** 10.3205/zma001081

**Published:** 2017-02-15

**Authors:** Franziska-Antonia Samos, Marcus Heise, Stephan Fuchs, Susanne Mittmann, Alexander Bauer, Andreas Klement

**Affiliations:** 1 Martin-Luther-University Halle-Wittenberg, Medical Faculty, Institute of General Practice and Family Medicine, Halle (Saale), Germany

**Keywords:** undergraduate medical education, general practice, evaluation, elective compulsory course, speciality-choice

## Abstract

**Background: **Primary health care in rural regions is currently undergoing a global crisis in respect of the next generation of practitioners. National and international recommendations advise placing greater emphasis upon practical skills and competences in medical studies. It is also in the interest of training the next generation to include mentoring and longitudinal integration of contact to teaching practices for general medicine in an early stage. Consequently, the *General Practice Class* (KAM) was introduced in Halle in 2011 as an elective with 20 individually mentored students per year, beginning with the first subject-related semester. We are now reporting on the results of the evaluation for the first two years.

**Method: **A standardised online survey was carried out with all students who took part in the KAM in the two years 2011 and 2012 (N=38). For both years the survey was made at the end of the first summer semester on the basis of an adapted version of the *Heidelberger Inventar zur Lehrevaluation (Heidelberg Inventory for the Evaluation of Teaching, HILVE-II)* and the *Berliner Evaluationsinstrument für selbsteingeschätzte, studentische Kompetenzen (Berlin Evaluation Instrument for the self-assessment of student competences, BEvaKomp)*. Furthermore, each year the preference for the choice of specialty and location of a medical practice was queried. Predictors for the preference of the chosen specialty and the location of a medical practice were estimated by binary logistic regression analysis. Via univariate evaluations the number of students who reported an increase in knowledge in different areas of competence as a result of the KAM was counted. Correlations between the intention to remain in the KAM and the quality of teaching were evaluated on the basis of bivariate correlations.

**Results:** 48% of the students agreed partly or fully that the KAM seminars enhanced their specialist competence. This individual acquiring of competence in the model project represented a significant predictor for the preferred choice of the area (OR 7.98; 95% CI [1.27-50.27], p=0.027). Students who assessed the commitment (r=0.504), support (r=0.526) and interaction management (r=0.529) of the mentors positively were more likely inclined to continue their participation in the KAM.

**Conclusion: **The successful conveyance of care-relevant competences to students proved to be an important predictor in our project for the preference of the specialty general practice. This requires that the medical mentors are suitably trained and that the students are specifically prepared for practical experience.

## Zusammenfassung

**Hintergrund: **Die medizinische Grundversorgung in ländlichen Regionen befindet sich weltweit in einer Nachwuchskrise. Internationale und nationale Empfehlungen raten zur Stärkung praktischer Fertigkeiten und Kompetenzen im Medizinstudium. Dazu gehört, auch im Interesse der Nachwuchsbindung, den Kontakt zu allgemeinmedizinischen Lehrpraxen frühzeitig, mentoriert und longitudinal zu integrieren. Dazu wird die *Klasse Allgemeinmedizin* (KAM) in Halle seit 2011 als Wahlpflichtfach mit 20 individuell mentorierten Studierenden pro Jahr beginnend mit dem ersten Fachsemester durchgeführt. Wir berichten über die Evaluationsergebnisse der ersten zwei Jahre.

**Methodik: **Eine standardisierte Online-Befragung wurde mit allen Teilnehmern der KAM in zwei Jahrgängen 2011 bis 2012 (N=38) durchgeführt. Die Befragung erfolgte jeweils zum Ende des ersten Sommersemesters und nutzte eine adaptierte Version des *Heidelberger Inventars zur Lehrevaluation* (HILVE-II) und das *Berliner Evaluationsinstrument für selbsteingeschätzte, studentische Kompetenzen* (BEvaKomp). Ferner wurde jährlich die Präferenz zu Fachgebietswahl und Ort der Niederlassung erfragt. Mittels binär-logistischer Regression wurden Prädiktoren für die Präferenz zur Fachgebietswahl Allgemeinmedizin und Ort der Niederlassung geschätzt. Durch univariate Auswertungen wurden die Anteile der Studierenden ausgezählt, die in verschiedenen Kompetenzbereichen einen Wissenszuwachs durch die KAM berichteten. Zusammenhänge zwischen den beabsichtigten Verbleib in der KAM und der Lehrqualität wurden anhand bivariater Korrelationen ausgewertet.

**Ergebnisse: **48% der Studierenden stimmten der Aussage eher oder völlig zu, dass die Seminare der KAM bei ihnen zu einer Steigerung der Fachkompetenz geführt haben. Dieser individuelle Kompetenzerwerb im Modellprojekt stellte einen signifikanten Prädiktor für die Präferenz zur Fachgebietswahl Allgemeinmedizin dar (OR 7,98; 95%-KI [1,27-50,27], p= 0,027). Studierende, die das Engagement (r=0,504), die Betreuung (r=0.526) und das Interaktionsmanagement (r=0,529) der Mentoren positiv einschätzten, waren eher gewillt, ihre Teilnahme an der KAM fortzusetzen.

**Schlussfolgerung: **Die erfolgreiche Vermittlung versorgungs-relevanter Kompetenzen an Studierende erwies sich in unserem Projekt als wichtigster Prädiktor einer Präferenz einer Fachgebietswahl Allgemeinmedizin. Hierfür müssen die ärztlichen Mentoren entsprechend geschult und Studierende gezielt auf die Praxiserfahrungen vorbereitet werden.

## Introduction

Primary health care in rural regions is currently undergoing a global crisis in respect of the next generation of practitioners. In Germany this applies primarily to the new federal states [1]. Around one half of the doctors currently practicing will presumably retire from professional life in the next 10 years due to age [2], [3]. Due to the insufficient number of students and young doctors interested in further education to a general practice specialist, in many locations, particularly in rural and structurally weak regions, it is expected that the requirement for general practitioners cannot be covered [4], [5]. For example, it has been very clearly shown for Saxony-Anhalt that mainly "natives" with a rural background are motivated to establish a rural medical practice there. An influx of young doctors from other federal states and with an urban background is not expected [5]. National and international recommendations advise placing greater emphasis upon core competences in the studies and the increased conveyance of primary care competences. In this respect, it appears to be particularly advisable to include mentoring and longitudinal integration of contact to teaching practices for general medicine in an early stage [1], [6].

In accordance with the recommendations of the Committee for Primary Health Care of the Gesellschaft für Medizinische Ausbildung (Association for Medical Education, GMA), in Halle (Saale) the educational project General Practice Class (KAM) was developed. Since autumn of 2011 it has been offered as an elective in the pre-clinical and clinical phases of study [6]. The KAM follows international models which, with similar curricular elements, have made a considerable contribution to ensuring rural health care as well as to the enhanced conveyance of primary health care – related competences in medical studies for up to 30 years [7], [8]. The generally accepted hypothesis of such initiatives is that the preferences for the (future) choice of speciality depend strongly on beginning as early as possible, on the time scope and the quality of the primary health care – related educational offering [9]. Beyond the academic offering, a comparable study was able to show that particularly medical lecturer with high professional satisfaction acted as a role model [10].The objective of the KAM is to increase the number of Halle medical students interested in establishing a practice as a general practitioner in rural regions for the long term by early, individual and mentored practice orientation.The present article gives an initial overview and educational evaluation of the KAM participants for the pilot phase of the 2011/12 winter semester to the 2013 summer semester. The questions were designed to examine

- Which competences the students name as having acquired in the KAM,
- Which factors are relevant for (subsequently) preferring a "general practitioner's" career path,
- Which factors influence the preference for the choice of location for a medical practice and
- Which factors influence the decision to continue with the KAM project.

## Methods

### Curriculum 

The curriculum of the KAM is comprised of three modules, which are offered each semester in two-hour seminars, focussing on the points skills training, medical case reflection and communication training [11], [12]. Skills training offers preparation for procedures in the practice, examination techniques and apparative methods. Medical case reflection offers room to discuss and reflect upon practical experiences. In communication training important capabilities for developing, improving and maintaining of the general practitioner – patient relationship are conveyed. Active and interactive teaching methods (practice-oriented role playing) furnish the basis for this. In addition to the seminars, the students take part in the so-called practice days in a general practice in the rural south of Saxony-Anhalt. The resident general practitioner furnishes support to “his” students during the entire course of studies as a mentor (1:1 support). The lecturer team consisted of two general practitioners from Saxony-Anhalt and a linguist. 

#### Random sample

Parallel to the implementation phase of the first two KAM years 2011 and 2012 all KAM participants, including those who dropped out, were queried. The survey took place at the end of the respective summer semester. Participation in the survey was voluntary. All data were gathered in pseudonymised form. As the length of participation varied between KAM 2011 and KAM 2012, the data were censored to the first year of KAM participation in order to obtain comparable data collection periods.

#### Materials

Data gathering took place by means of online questionnaires. The basis for the survey was the *Berliner Evaluationsinstrument für selbsteingeschätzte, studentische Kompetenzen (Berlin Evaluation Instrument for the self-assessment of student competences, BEvaKomp)* [13]. The instrument acquires the self-assessed improvement of the students in the categories professional competence, methodological competence, communication competence and practical competence. The students were asked to evaluate their personal competence on a scale from “1” (not applicable) to “5” (fully applicable). For the evaluation of educational quality an adapted version of the *Heidelberger Inventar zur Lehrevaluation (Heidelberg Inventory for the Evaluation of Teaching, HILVE-II)* [14] was used. The instrument acquires the interest of the students in the seminar topics, the subjective assessment of the educational benefit and evaluates the activities of the lecturer, composing a mean index with a range of values from “1” (not applicable) to “5” (fully applicable). 

#### Endpoints

The primary endpoint of the study was the identification of factors which motivated the students to (subsequently) prefer a career path as a “general practitioner”. The secondary endpoint was the investigation of factors for the preference of a (future) practice in a rural region, the acquiring of competence by participation in the KAM seminars and factors favouring continuing with the model project. The acquiring of competence was operationalised as a pooled score in the areas of communication, methods, professional and practical competence of the BEvaKomp. The overall assessment of the educational quality was mirrored in the mean values for the partial areas communication training, medical case reflection and skills training of the HILVE-2. 

#### Statistical Analysis

The influence of the mean subjective improvement in competence within the scope of the model project upon the choice of the subsequent further specialist education was estimated in binary logistical regression. Likewise, socio-demographic attributes as predictors for the choice of practice location were used in binary logistic regression. In both analyses odds ratios and their related 95% confidence intervals were estimated. Within the scope of univariate evaluations the number of students reporting an enhancement of knowledge in different areas of competence as a result of the model project was counted.

The overall assessment of the educational quality was calculated for each semester from the mean values of the main dimensions of the HILVE-2 and then correlated with the self-reported intent to continue taking part in the project. Here a significance threshold of α=0.05 was defined.

## Results

A total of N=38 students from the KAM years 2011 and 2012 were included in the study. Table 1 (Tab. 1) gives the socio-demographic attributes for both years (see Table 1 (Tab. 1)).

The majority of those taking part were between 20 and 30 years old and came from regions of rural character. In each case, one half of the participant already had contact to general medicine before beginning the study, whether via the medical practice of their parents or professional education in the medical sector. Around two thirds of the students taking part were women, corresponding to the distribution by sex of the first semester students in Halle. Noticeable in the combined years is the predominantly younger age group and parents in the medical profession in 2011 (see Table 1 (Tab. 1)). Of the 38 participants at the start 14 (37%) did not continue with the project (mostly “regular” following the medical preliminary examination). None of those who dropped out had completed a former education in the medical profession before the study, compared with 11 who had a former education in the medical profession out of 24 continuing with the study (p=0.015 (bilateral)).

### Acquisition of competence via the KAM seminars 

**Figure 1:**
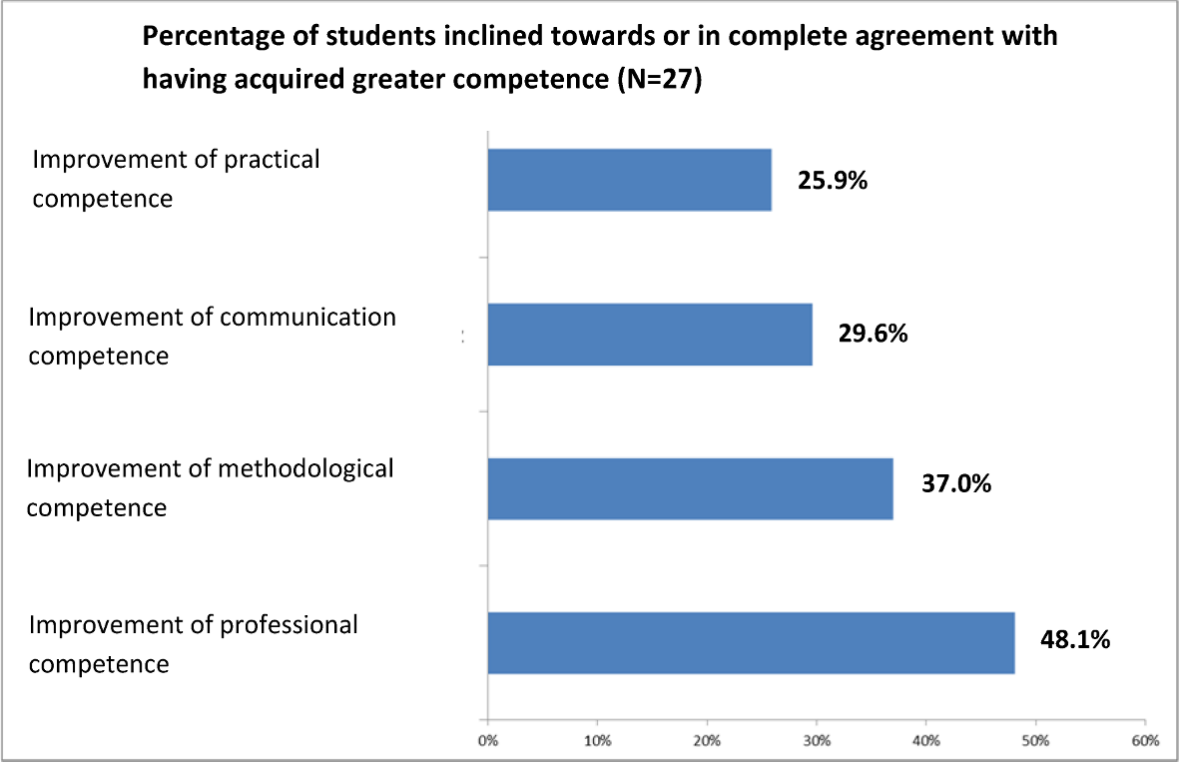
Percentage of students that assessed their acquired competences with the categories “4” or “5” on a scale of “1” (not applicable) to “5” (fully applicable) in the BEvaKomp [13]

With the BevaKomp instrument [13] the students were asked to name the areas in which they subjectively experienced enhanced competence as a result of taking part in the seminars. The number of students inclined towards or in complete agreement with the items after taking part one year in the model project versus those inclined towards or completely rejecting the items was determined. 48.1% of the students were inclined towards or in complete agreement that the KAM seminars enhanced their professional competence. More than one third of the participants (37%) subjectively experienced an improvement in methodological competence. 26% and 30% reported improvement in practical and communication competence (see Figure 1 (Fig. 1)). 

#### Factors for (subsequently) preferring a "general practitioner's" career path 

In the logistic regression model the mean health care – oriented competences determined in the BEvaKomp significantly influence the “general practitioner's” career path (see Table 2 (Tab. 2)).

For each point by which the self-assessed competence of the students increases as a result of the seminars the probability of wanting to become a "general practitioner" increases by nearly a factor of eight (OR 7.98; 95% CI [1.27-50.27], p=0.027). 

#### Factors for (subsequently) preferring to practice in a rural area

Complementary to the items of the BEvaKomp the students were asked to indicate whether they intended to practice in a rural region following their continued specialist medical education. As influencing factors the sex, the origin of the students and the profession of their parents were examined (see Table 3 (Tab. 3)).

No significant influence on the preference for practicing in a rural region could be determined for any of the predictors employed. Overall, the observations can also not be generalised (see Table 3 (Tab. 3)). 

#### Continuance in the KAM model project

On the basis of the students' evaluation of the seminars in the categories of the HILVE-II it is apparent that the probability of continuing with the project increase when the students assess both the lecturers and the seminars positively (see Table 4 (Tab. 4)).

The stated intention to continue taking part in the KAM model project correlates significantly with a better evaluation of the perceived commitment of the lecturers (r=0.504; p=0.007), with subjectively better perceived support of the lecturers (r=0.526; p=0.005) and with a positively perceived interaction management (r=0.529; p=0.005). At the same time, no significant correlation was found in respect of the structure of the seminars, the depth of involvement with the content of the lectures, the didactic competence of the lecturers, the interest of the students in the topics presented, the educational benefit or the form of instruction chosen (communicative-activating versus frontal) (see Table 4 (Tab. 4)). 

## Discussion

Following the two-year pilot phase of the “General Practice Class” more than 60% of the participants have chosen to continue the project, whereby the quality of support, the commitment of the lecturers and the interaction in the project appear to be of importance. The subjective acquisition of health care – relevant competences in the KAM appears to have a significant influence on the (subsequent) preference for a “general practitioner's” career path.

The strong correlations between the students' (subsequent) preference for a general practitioner career path and the commitment of the lecturers, as well as the intensity of support and the interaction management in the project once again support the interpretation that a positive role model by the mentors is largely responsible for this [10], [15]. Currently ongoing international studies show that, in spite of positive educational benefits, even elaborately designed and realised curricular interventions have less influence upon the choice of medical specialty and the location of a practice than personal “practical experiences” [16], [17]. Accordingly, mentors are an indispensable part of similar projects [7], [8] and are particularly recommended for sitting in on lectures in the early phases of study, for which both students and mentors must however be prepared [6], [18], [19].

Although socio-demographic factors (such as origin, partner or children) have frequently been mentioned as predictors for (subsequently) preferring to practice in a rural area, in our survey sampling we found no evidence of such correlations [5], [15], [20]. This could be due, for example, to the limited number of random samples and/or the self-selection of the students for participation in the project by comparison with the “complete surveys” covering several years [5]. 

In respect of professional development and the choice of medical specialty, mentoring programmes for students are successful and meaningful in different medical specialties [18], [19] and are urgently wished, even by emerging (general) practitioners within the scope of their continuing education [21]. Accordingly, our initial results largely confirm those of publications of overview character which describe experiences from similar educational projects with peer group character [10], [15]. A current German cross-sectional survey showed similar effects favouring a general practitioner's career path as a result of taking part in general medical educational projects [20].

### Strengths and weaknesses

The present article is the first of its kind that reports on the course of implementation of an educational project for the furtherance of a rural doctor's identity formation amongst medical students. The limitations of the study are the limited survey sample size and a possible selection bias. In particular, a possible systematic distortion of the results due to the self-selection of the KAM students is conceivable: whoever is interested in pursuing general medicine at the beginning of studies in Halle may well prefer the KAM as an elective and accordingly state a preference for the “general practitioner's” career path – possibly without being influenced by the educational project itself [15], [20]. Furthermore, distortion effects as a result of “social desirability” cannot be excluded amongst the students who continue in the project. In spite of the inconspicuous selection of the participants and the limited survey sample size, however, the observed effects allow one to derive impulses for the content design and further development of such projects.

#### Result and outlook

The fifth year of the General Practice Class began in the 2015/16 winter semester. A total of 82 students are currently taking part in the KAM (KAM 11:10 students, KAM 12:7 students (+10 “latecomers” following the preliminary medical examination), KAM 13:15 students (+5 “latecomers”), KAM 14:19 students, KAM 15:20 students). 70 rural doctor mentors from southern Saxony-Anhalt support the project. The number of applicants has levelled off at around 17 % of the average number of 230 students in the first semester in Halle (KAM 11:N=40, KAM 12:N=19, KAM 13:N=25, KAM 14:N=37, KAM 15:N=42). In the selection interviews for filling the annual 20 places in the project, many students mention “[…] positive reports from informal contacts with students of earlier years […]” as a reason for applying. In addition, the participants were encouraged by (trans-) regional attention in the media (e.g. Deutschlandfunk and ZEIT-Campus), as well as official recognition (e.g. “Land of Ideas” in 2014: winner of the national prize in the category education; Saxony-Anhalt Demography Prize in 2015). Within the faculty the “functioning” of the project has contributed to greater esteem for the General Practice Section and to a stronger health care – relevant orientation of the teaching content and methods (e.g. in the skills lab). This furnishes support for the outwardly perceived regional reputation and image of the faculty. 

Although the KAM project was shown to be viable and – judging from the number of applicants and participants, as well as the echo in the media – with limited resources (1.0 “researcher position”) successful, at the present time we have no proof of its effectiveness. The same is true of the subjective enhancement of competence, which must first prove itself in practical work. This will only be reflected in long-term observations, e.g. when beginning the next phase of education for the students of the first KMA year, commencing in 2017. In future evaluations we will employ an in the meantime validated instrument for the choice of medical specialty [22] and also improve the effects of individual curriculum and project sections, such as an appreciative attitude towards primary care areas, taking account of greater differentiability – in order to improve the comparability of similar projects as well. 

## Competing interests

The authors declare that they have no competing interests.

## Figures and Tables

**Table 1:**
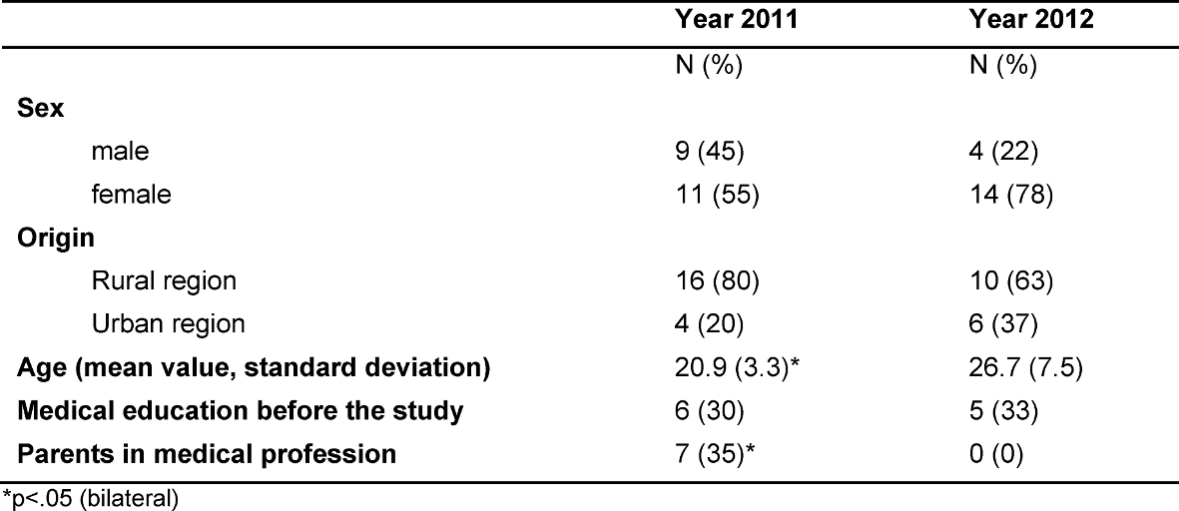
Socio-demographic attributes for the project participants of KAM years 2011 and 2012

**Table 2:**
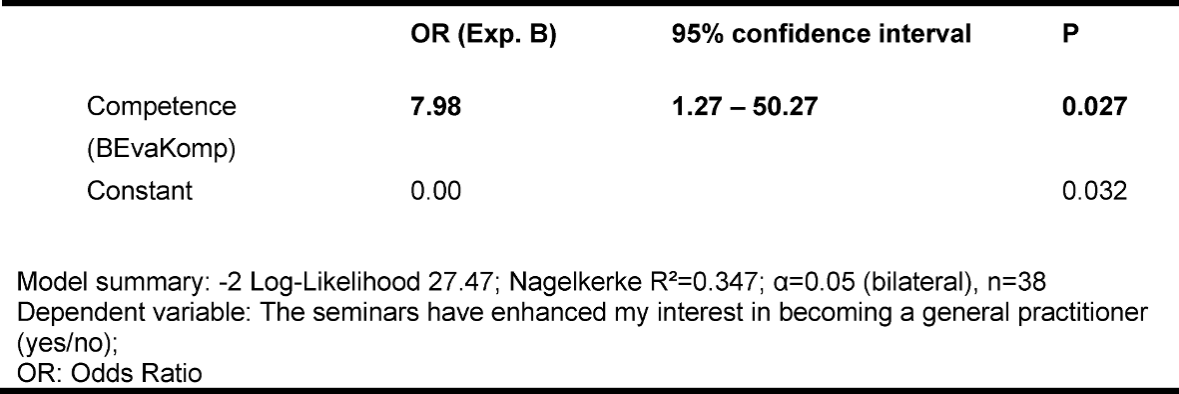
Binary-logistic regression model for the subsequent preference of a “general practitioner's” career path

**Table 3:**
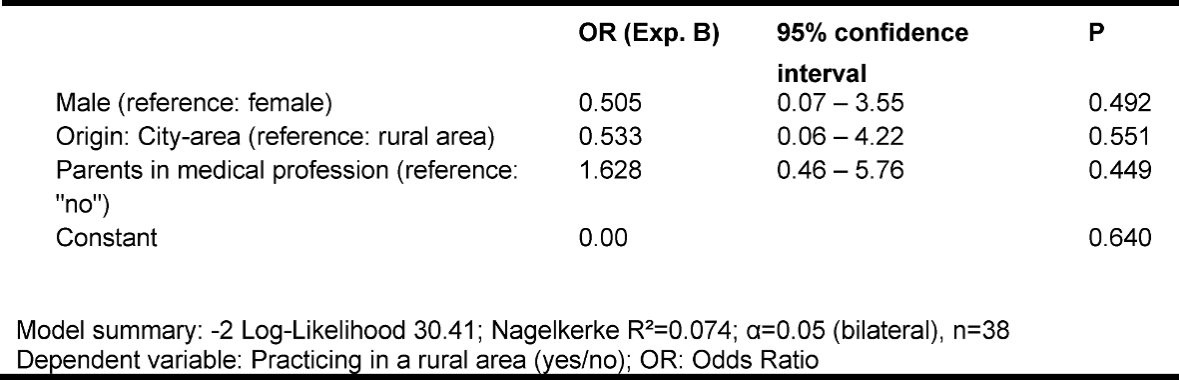
Binary-logistic regression model for the subsequent preference of a practice in a rural area

**Table 4:**
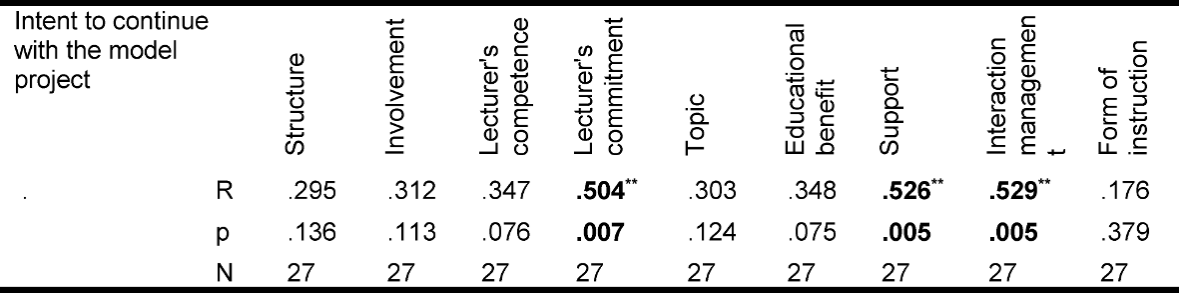
Correlations between the students' evaluation of the educational quality (HILVE-II) in the project and the intention to continue taking part in the KAM. Significant correlations are emphasised.*
